# Editorial: Advances and challenges in orbital imaging: from radiography to modern MRI and 3D technologies

**DOI:** 10.3389/fopht.2026.2005013

**Published:** 2026-10-26

**Authors:** Alon Kahana

**Affiliations:** 1 Department of Ophthalmology, Oakland University William Beaumont School of Medicine, Rochester, MI, United States; 2 Kahana Oculoplastic and Orbital Surgery, Livonia, MI, United States

**Keywords:** computed tomography, magnetic resonance imaging, ocular surface squamous neoplasia, orbital imaging, thyroid eye disease, vascular malformations

Orbital imaging has progressed from radiographs to computed tomography (CT), magnetic resonance imaging (MRI), and techniques that characterize tissue activity and vascular flow. The seven contributions in this Research Topic examine thyroid eye disease, orbital masses, vascular anomalies, lacrimal gland disease, and ocular surface neoplasia. Together, these articles emphasize that diagnostic value depends on matching the modality to the clinical question, standardizing measurements, and interpreting findings in context.

Two contributions address thyroid eye disease (TED). Roelofs et al. review single-photon emission computed tomography combined with CT (SPECT/CT), particularly technetium-99m diethylenetriaminepentaacetic acid imaging, as a potential marker of orbital inflammatory activity. Uptake measurements and radiomics models show promise, but acquisition timing, reference regions, and measurement methods affect comparability. Moreover, SPECT/CT studies that use clinical activity score (CAS) as their reference standard cannot independently establish superiority over that score. Still, this technique has promise as a research tool and for challenging cases. Potvin et al. review MRI and serologic biomarkers. Short tau inversion recovery sequences and diffusion-weighted imaging can identify changes associated with inflammation; apparent diffusion coefficient (ADC) measurements offer quantitative information. These approaches may complement CAS, but require further validation and standardization.

CAS remains clinically useful, yet it does not fully capture TED heterogeneity. Background literature on disease phenotypes (1) highlights the distinction between visible inflammatory signs and the underlying orbital process. Congestion and inflammation can coexist, and a low CAS does not exclude clinically important disease. Subramanian et al. (2) review responses to teprotumumab in long-duration TED, challenging the assumption that longstanding disease is necessarily unresponsive to medical therapy. The “white eye” phenotypes further illustrate the distinction between activity and severity. Uddin and Hamed Azzam (3) describe apical crowding and dysthyroid optic neuropathy despite low CAS, as well as predominantly fat-associated expansion with few inflammatory signs. Lastly, Lee et al. (4) report ethnic variation in clinical and imaging features, including shorter intervals between thyroid diagnosis and TED in Asian and Hispanic patients than in non-Hispanic White patients within their cohort. These findings reveal ethnic variations in TED signs and symptoms.

Imaging localizes posterior crowding and distinguishes patterns of muscle and fat enlargement that an activity score cannot describe. It complements clinical assessment of optic nerve function; it does not replace it. Similarly, improvement in external inflammatory signs does not guarantee resolution of apical compression. The aim is to integrate activity, anatomy, and functional risk rather than ask any single measure to represent the entire disease.

Levanon et al. and Fernández et al. approach orbital masses through complementary CT and MRI perspectives. Enhancement patterns, fat content, bone changes, lesion location, and diffusion characteristics help narrow the differential diagnosis. Tendon appearance is a useful example of the limits of individual signs: tendon thickening occurs in fewer than half of reported orbital myositis cases, so tendon sparing does not exclude myositis (7). Low ADC values may support a diagnosis of lymphoma, but overlap with other lesions limits universal thresholds (8). Rapid growth of a pediatric orbital mass should raise concern for rhabdomyosarcoma; growth rate alone is not diagnostic. Imaging guides further evaluation and, when indicated, tissue diagnosis.

Arjomandi et al. demonstrate why vascular imaging must reflect lesion physiology. Dynamic angiographic techniques characterize arterial inflow and venous drainage, while imaging during a Valsalva maneuver can reveal distensible venous components that are inconspicuous at rest. Conventional MRI helps define the extent and internal characteristics of lymphatic-venous malformations. Modality selection therefore depends on the suspected flow pattern, lesion extent, and treatment being considered. A static image may answer an anatomical question while leaving a hemodynamic question unresolved.

Caltabiano et al. extend this reasoning to lacrimal gland disease. Epithelial tumors, lymphoid lesions, and inflammatory disorders share imaging features, so margins, bone remodeling or destruction, diffusion, and patterns of extension must be assessed together. A wedge sign can occur in both malignant and nonmalignant conditions; its significance depends on the lesion category and clinical setting. Diffusion restriction can occur in inflammatory disease, and no single sign establishes malignancy. Clinical correlation remains essential when imaging patterns overlap.

Fogel Levin et al. examine anterior segment optical coherence tomography (AS-OCT) and ultrasound biomicroscopy (UBM) in ocular surface squamous neoplasia. AS-OCT provides high-resolution epithelial detail, whereas UBM offers greater penetration for assessing deeper structures. Their review reports axial resolutions of 2–7 µm for AS-OCT and approximately 25 µm for UBM, with respective penetration depths of 2–3 mm and 4–5 mm; performance depends on the device and lesion. Thick or keratinized lesions can obscure deeper tissue on AS-OCT, making complementary UBM valuable when scleral or intraocular extension is suspected.

Across these seven contributions, improved technology broadens what can be measured but does not replace the need for clinical judgment. Quantitative biomarkers require reproducible acquisition and analysis; diagnostic patterns require correlation with clinical findings and, where appropriate, pathology. Future work should prioritize multicenter validation, clinically meaningful outcomes, and practical implementation alongside technical innovation. Artificial intelligence and radiomics may assist interpretation, but their usefulness must be demonstrated across institutions and patient populations. The central challenge is to translate increasingly detailed images into dependable decisions for individual patients.
